# Supplementary material for: Amorphization of Ethenzamide and Ethenzamide Cocrystals—A Case Study of Single and Binary Systems Forming Low-Melting Eutectic Phases Loaded on/in Silica Gel
Source: Pharmaceutics. 2023 Apr 13;15(4):1234. doi: 10.3390/pharmaceutics15041234 (PMC10142476; doi:10.3390/pharmaceutics15041234)
Supplement: Supplementary file 1 [file pharmaceutics-15-01234-s001.zip › pharmaceutics-2305249-supplementary.pdf]

Supplementary Materials

Amorphization of enthenzamide And Enthenzamide Cocrystals —A Case Study Of Single And Binary Systems Forming Low-Melting Eutectic Phases Loaded On/In Silica Gel

Katarzyna Trzeciak, Ewelina Wielgus, Sławomir Kaźmierski, Tomasz Pawlak
and Marek J. Potrzebowski*.

Centre of Molecular and Macromolecular Studies Polish Academy of Sciences, Sienkiewicza 112, 90-363 Lodz, Poland

*Corresponding author: marek.potrzebowski@cbmm.lodz.pl

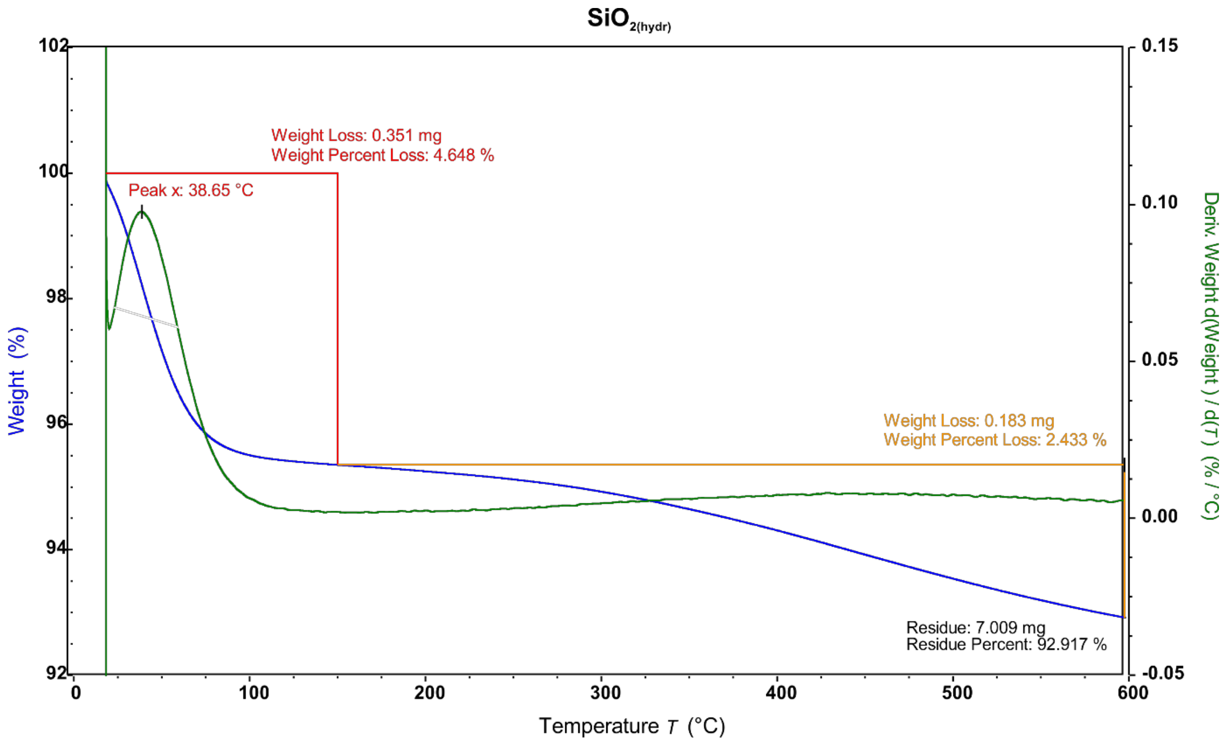

Figure S1. TGA curve of $SiO_{2(hydr)}$.

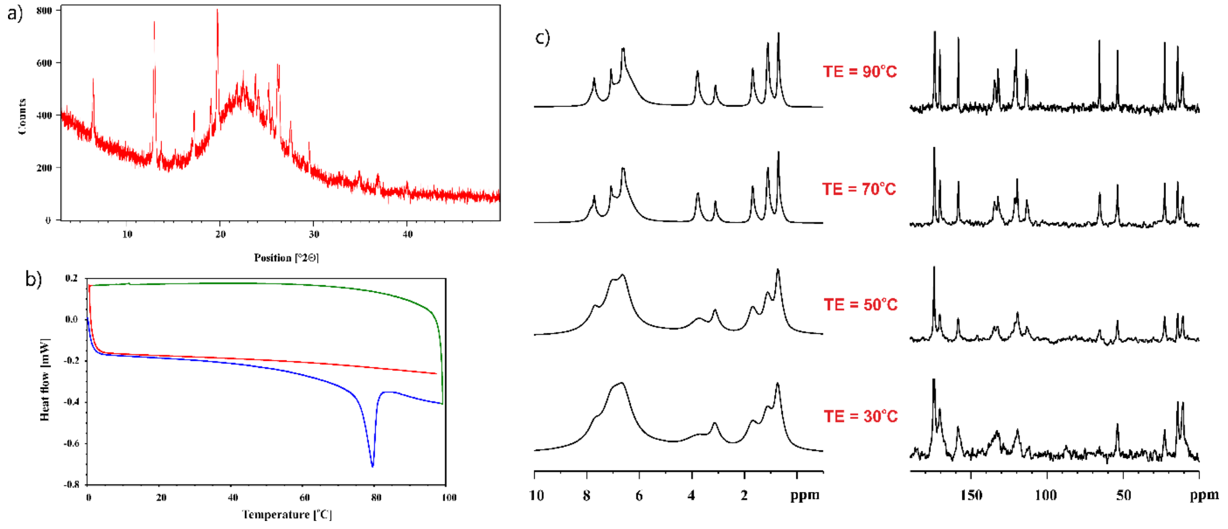

Figure S2. (**a**) PXRD recorded on a PANalytical 3 kW system in Bragg Brentano geometry and with a Cu Kα (λ = 1.5425 Å) source, (**b**) The DSC measurement. The first heating run from 0°C to 100°C (blue line), then a cooling run from 100°C to 0°C (green line), followed by a second heating run from 0°C to 100°C (red line), (**c**) VT NMR data ^{1}H MAS (left column) and ^{13}C CP MAS (right column) recorded with spinning rate of 12 kHz for a physical mixture of ET:EMA/$SiO_{2(hydr)}$.

Table S1. Cumulative release (%) of ET for all analyzed samples (water, pH 5.7).

	ET	ET:GLU	ET:EMA	ET:SiO_2/MM	ET:GLU/SiO_2/MM	ET:EMA/SiO_2/MM
**Time (min)**	**Cumulative release (%)**					
0	0	0	0	0	0	0
1	1.28	5.16	3.63	2.56	7.84	8.50
2	7.05	12.26	5.89	35.90	26.15	49.68
4	23.73	25.83	10.47	73.12	69.97	60.85
6	43.63	34.89	15.71	83.46	83.12	75.95
8	51.37	41.38	22.92	86.75	87.14	81.26
10	63.60	45.94	28.82	90.06	89.19	85.27
15	80.98	54.38	41.93	93.36	91.25	89.94
20	86.20	63.47	52.43	93.46	93.97	92.66
30	93.35	73.22	66.87	96.13	96.03	96.68
40	95.37	82.98	76.09	96.88	96.14	96.79
50	96.12	87.58	83.37	97.63	96.90	98.20
60	96.87	91.55	88.03	99.02	97.66	98.96
75	97.61	95.52	94.01	99.13	97.77	99.73
90	98.36	96.92	97.38	98.59	96.57	98.53
105	98.47	98.96	100.11	99.98	98.63	99.94

	ET/SiO_2/TM	ET:GLU/SiO_2/TM	ET:EMA/SiO_2/TM	ET/SiO_2/BM	ET:GLU/SiO_2/BM	ET:EMA/SiO_2/BM
**Time (min)**	**Cumulative release (%)**					
0	0	0	0	0	0	0
1	7.69	10.87	31.34	12.14	21.68	35.42
2	13.09	19.58	47.05	39.30	37.79	40.32
4	21.56	27.57	53.07	54.34	46.92	45.22
6	38.51	32.67	55.37	59.40	51.87	49.44
8	46.24	37.06	56.92	62.33	53.32	51.58
10	57.83	42.90	60.72	62.40	57.58	55.11
15	64.82	49.46	65.26	67.46	61.14	59.33
20	71.82	56.04	67.57	70.40	65.40	63.57
30	77.28	61.90	75.11	75.47	71.77	68.50
40	78.90	66.32	81.16	77.70	75.34	73.44
50	80.53	71.46	85.73	81.36	78.22	76.99
60	81.39	75.89	89.56	82.16	81.11	79.16
75	80.71	81.05	93.39	84.39	82.59	83.41
90	80.80	87.66	98.71	85.92	86.88	85.59
105	80.89	92.83	100.32	87.44	89.07	88.46

Table S2. Cumulative release (%) of ET for all analyzed samples (simulated gastric fluid without pepsin (SGFsp), pH 1.2).

	ET	ET:GLU	ET:EMA	ET:SiO_2/MM	ET:GLU/SiO_2/MM	ET:EMA/SiO_2/MM
**Time (min)**	**Cumulative release (%)**					
0	0	0	0	0	0	0
1	0.90	6.06	2.84	2.06	9.78	6.18
2	2.67	11.22	6.28	35.30	29.50	17.19
4	9.46	22.70	18.18	56.82	55.03	35.25
6	18.49	33.49	25.10	81.08	74.86	44.75
8	27.12	40.22	29.12	86.09	84.62	54.38
10	36.44	48.08	36.90	90.08	92.65	65.93
15	57.00	56.20	47.91	91.39	93.79	83.86
20	70.20	65.21	56.72	94.06	94.67	92.85
30	86.39	77.67	69.98	95.97	96.09	95.90
40	91.87	84.25	78.67	97.83	96.38	99.50
50	91.39	88.88	86.69	97.75	98.44	98.98
60	93.22	91.85	91.94	97.27	97.32	98.77
75	93.26	95.40	95.47	99.07	98.20	99.45
90	96.33	98.31	99.38	99.39	99.17	99.71
105	96.00	97.49	99.38	99.35	98.27	98.81

	ET/SiO_2/TM	ET:GLU/SiO_2/TM	ET:EMA/SiO_2/TM	ET/SiO_2/BM	ET:GLU/SiO_2/BM	ET:EMA/SiO_2/BM
**Time (min)**	**Cumulative release (%)**					
0	0	0	0	0	0	0
1	16.19	18.72	43.88	7.02	20.13	24.08
2	28.57	24.77	54.76	39.25	27.91	36.35
4	45.03	28.80	60.19	59.28	36.03	46.51
6	51.22	34.87	64.40	63.02	41.22	53.02
8	54.72	37.30	64.92	65.98	46.92	57.96
10	57.04	40.88	68.22	67.59	50.66	60.09
15	62.27	45.63	74.50	73.21	59.26	69.50
20	67.37	51.61	77.67	76.31	64.07	72.60
30	70.88	62.80	84.88	81.09	70.90	79.45
40	75.34	69.22	88.52	83.78	76.57	84.16
50	81.37	74.67	91.18	85.12	80.28	89.30
60	84.59	80.20	94.25	86.52	84.63	91.34
75	85.85	86.94	94.62	89.78	87.30	94.38
90	86.69	88.73	97.84	88.45	91.26	96.92
105	87.24	95.90	97.87	90.87	93.50	98.06
